# An insight of rapamycin against cadmium’s neurotoxicity

**DOI:** 10.18632/oncotarget.14872

**Published:** 2017-01-28

**Authors:** Chong Xu, Shile Huang, Long Chen

**Affiliations:** Jiangsu Key Laboratory for Microbes and Functional Genomics, Jiangsu Key Laboratory for Molecular and Medical Biotechnology, College of Life Sciences, Nanjing Normal University, Nanjing, P. R. China

**Keywords:** rapamycin, cadmium, ROS, neuronal cells, PP2A, JNK, Erk1/2, Neuroscience

Cadmium (Cd), a toxic environmental pollutant, has a long biological half-life (15–20 years) and thus can be readily accumulated in human body with age. Due to its high permeability to blood-brain barrier, Cd can evoke overproduction of reactive oxygen species (ROS) and consequently induce neuronal cell death in brain. Cd has been regarded as a possible etiological factor for human neurodegenerative diseases, such as Parkinson’s disease, Alzheimer’s disease, and Huntington’s disease [1]. Thus, it is of great importance to find effective interventions against Cd-induced oxidative stress in the central nervous system.

Rapamycin is a potent and specific inhibitor of mammalian/mechanistic target of rapamycin (mTOR) [2]. Abundant evidence suggests that rapamycin may possess neuroprotective effect on various neurological diseases [2]. Our recent studies have revealed that pretreatment with rapamycin prevents Cd-induced neuronal cell death, in part by attenuating Cd-induced ROS activation of mTOR signaling pathways *in vitro* and *in vivo* [3–5]. In addition, we have noticed that Cd-induced ROS can activate c-Jun N-terminal kinase (JNK) and extracellular signal-regulated kinase 1/2 (Erk1/2), partly contributing to neuronal apoptosis [6]. It has been shown that rapamycin impacts the activity of JNK and Erk1/2 under different conditions [7]. This prompted us to study whether and how rapamycin mitigates Cd neurotoxicity by preventing ROS from activation of JNK and Erk1/2 pathways.

The mitochondrion is not only the factory of energy but also the main generator of ROS in a cell. First of all, we investigated whether rapamycin prevents Cd from inducing ROS in the mitochondria of neuronal cells. For this, we employed thenoyltrifluoroacetone (TTFA), a mitochondrial complex II ubiquinone site inhibitor that blocks electron supply to ubiquinol and consequentially limits the formation of ubisemiquinone, and antimycin A, a mitochondrial complex III inhibitor that increases the lifetime of ubisemiquinone. Our results showed that co-treatment with rapamycin/TTFA inhibited Cd-evoked ROS more potently than treatment with rapamycin or TTFA alone in PC12 cells and mouse primary neurons, whereas treatment with antimycin A alone markedly triggered ROS, and further enhanced Cd-induced ROS, which could be repressed by rapamycin pretreatment in the cells [8]. Furthermore, Mito-TEMPO, a mitochondria-targeted antioxidant, significantly strengthened the inhibitory effects of rapamycin on Cd-induced ROS and neuronal apoptosis [8]. Hence, our results indicate that rapamycin attenuates Cd-induced neuronal apoptosis indeed by preventing Cd-elicited mitochondrial ROS.

Next, we tested whether rapamycin attenuates Cd neurotoxicity by blocking Cd-induced mitochondrial ROS activation of JNK and Erk1/2 pathways. Our data showed that rapamycin remarkably suppressed Cd-induced phosphorylation of JNK and Erk1/2 in PC12 cells and primary neurons, which was strengthened by addition of Mito-TEMPO [8]. To validate the involvement of JNK and Erk1/2 in rapamycin’s protection against Cd-induced neuronal apoptosis, SP600125 (JNK inhibitor) and U0126 (MEK1/2 inhibitor) were utilized. We found that SP600125 and U0126, respectively, potentiated rapamycin’s prevention from Cd-induced cell apoptosis [8]. Consistently, using recombinant adenoviruses expressing dominant negative c-Jun (dn-c-Jun) or MKK1 (MKK1-K97M), we observed that ectopic expression of dn-c-Jun or MKK1-K97M also potently improved the inhibitory effect of rapamycin on Cd neurotoxicity [8]. Furthermore, pretreatment with SP600125 or U0126, or expression of dn-c-Jun or MKK1-K97M enhanced the inhibitory effect of rapamycin or Mito-TEMPO on Cd-induced ROS [8]. Collectively, these results strongly suggest that rapamycin blocks Cd-induced mitochondrial ROS activation of JNK and Erk1/2 pathways, thereby rescuing against neuronal apoptosis triggered by Cd.

Finally, we studied how rapamycin prevents Cd-induced mitochondrial ROS from activating JNK and Erk1/2 pathways. It is known that PP2A negatively regulates JNK and Erk1/2, in response to stress response [6], and PP2A can be activated by rapamycin [7]. So, we examined whether rapamycin blocks Cd activation of JNK and Erk1/2 pathways by preventing Cd-induced mitochondrial ROS from inactivation of PP2A. The results showed that Cd-induced elevation of demethylation and phosphorylation of PP2A catalytic subunit, two events related to PP2A inactivation, was substantially suppressed by rapamycin or Mito-TEMPO alone, and more potently inhibited by their co-treatment in PC12 cells and primary neurons [8]. To corroborate the above findings, genetic recue experiments for PP2A were carried out. We observed that over-expression of wild-type PP2A reinforced rapamycin or Mito-TEMPO suppression of activated JNK and Erk1/2 pathways, as well as ROS production and apoptosis in PC12 cells in response to Cd [8]. Therefore, these data support that rapamycin ameliorates Cd-induced neuronal apoptosis by preventing mitochondrial ROS inactivation of PP2A, thus blocking activation of JNK and Erk1/2 cascades.

**Figure 1:**
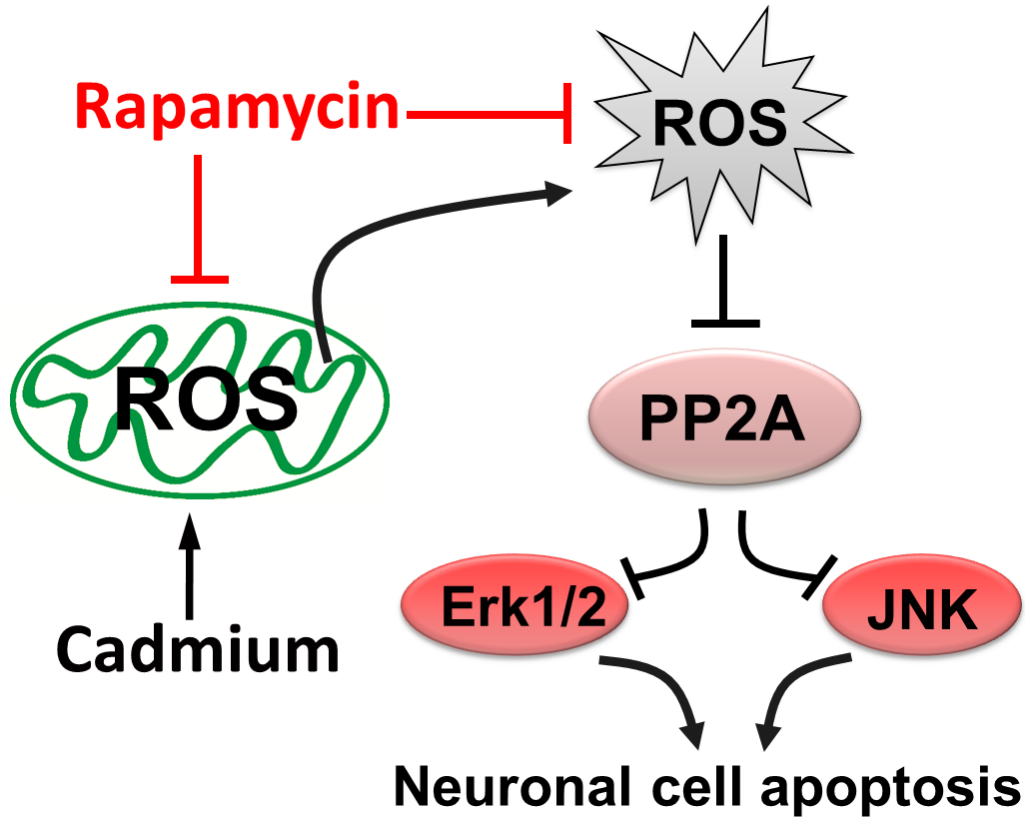
Schematic diagram of rapamycin against cadmium’s neurotoxicity Rapamycin prevents Cd-evoked mitochondrial ROS inactivation of PP2A, thus suppressing activation of JNK and Erk1/2 pathways, and rescuing neuronal cell apoptosis.

In conclusion, we have demonstrated that rapamycin prevents Cd-evoked mitochondrial ROS inactivation of PP2A, thus suppressing activation of JNK and Erk1/2 pathways, and rescuing neuronal apoptosis (Figure 1). The new finding highlights rapamycin as a promising agent for prevention of Cd-induced oxidative stress and neurodegenerative diseases.
